# Does vitamin-D intake during resistance training improve the skeletal muscle hypertrophic and strength response in young and elderly men? – a randomized controlled trial

**DOI:** 10.1186/s12986-015-0029-y

**Published:** 2015-09-30

**Authors:** Jakob Agergaard, Jeanette Trøstrup, Jacob Uth, Jonas Vestergard Iversen, Anders Boesen, Jesper L. Andersen, Peter Schjerling, Henning Langberg

**Affiliations:** Institute of Sports Medicine Copenhagen, Department of Orthopedic Surgery M, Bispebjerg Hospital and Center for Healthy Aging, Faculty of Health and Medical Sciences, University of Copenhagen, Copenhagen, Denmark; CopenRehab, Section of Social Medicine, Department of Public Health and Center for Healthy Aging, Faculty of Health and Medical Sciences, University of Copenhagen, Copenhagen, Denmark

**Keywords:** Vitamin-D, Resistance exercise, Skeletal muscle, Fiber type, Vitamin-D receptor

## Abstract

**Introduction:**

Recent studies have shown that vitamin-D intake can improve skeletal muscle function and strength in frail vitamin-D insufficient individuals. We investigated whether vitamin-D intake can improve the muscular response to resistance training in healthy young and elderly individuals, respectively.

**Methods:**

Healthy untrained young (*n* = 20, age 20–30) and elderly (*n* = 20, age 60–75) men were randomized to 16 weeks of daily supplementary intake of either 48 μg of vitamin-D + 800 mg calcium (Vitamin-D-group) or 800 mg calcium (Placebo-group) during a period and at a latitude of low sunlight (December-April, 56°N). During the last 12 weeks of the supplementation the subjects underwent progressive resistance training of the quadriceps muscle. Muscle hypertrophy, measured as changes in cross sectional area (CSA), and isometric strength of the quadriceps were determined. Muscle biopsies were analyzed for fiber type morphology changes and mRNA expression of vitamin-D receptor (VDR), cytochrome p450 27B1 (CYP27B1) and Myostatin.

**Results:**

In the vitamin-D groups, serum 25(OH)D concentration increased significantly and at week 12 was significantly different from placebo in both young men (71.6 vs. 50.4 nmol/L, respectively) and elderly men (111.2 vs. 66.7 nmol/L, respectively). After 12 weeks of resistance training, quadriceps CSA and isometric strength increased compared to baseline in young (CSA *p* < 0.0001, strength *p* = 0.005) and elderly (CSA *p* = 0.001, strength *p* < 0.0001) with no difference between vitamin-D and placebo groups. Vitamin-D intake and resistance training increased strength/CSA in elderly compared to young (*p* = 0.008). In the young vitamin-D group, the change in fiber type IIa percentage was greater after 12 weeks training (*p* = 0.030) and Myostatin mRNA expression lower compared to the placebo group (*p* = 0.006). Neither resistance training nor vitamin-D intake changed VDR mRNA expression.

**Conclusion:**

No additive effect of vitamin-D intake during 12 weeks of resistance training could be detected on either whole muscle hypertrophy or muscle strength, but improved muscle quality in elderly and fiber type morphology in young were observed, indicating an effect of vitamin-D on skeletal muscle remodeling.

**Trial registration:**

ClinicalTrials with nr. NCT01252381

**Electronic supplementary material:**

The online version of this article (doi:10.1186/s12986-015-0029-y) contains supplementary material, which is available to authorized users.

## Introduction

During recent years increased attention has been drawn to the influence of vitamin-D on the development of a variety of diseases. A relationship between vitamin-D insufficiency and osteoporosis as well as increased risk of bone fractures has been established [1–4]. Furthermore, low vitamin-D levels are connected to the development of diabetes [5], cardiovascular diseases [3, 6], cancer [3, 7], depression [3], osteoarthritis [8], multiple sclerosis [9] and maintenance of a healthy immune system [10], although the specific mechanisms have not been fully elucidated.

It is well known that both muscle strength and vitamin-D levels decrease with age [1, 11]. Low vitamin-D levels are associated with decreased muscle strength and poor physical function in elderly individuals [12]. In addition, it has been proposed that vitamin-D plays an important role for obtaining optimal skeletal muscle function [13, 14].

Expression of the vitamin-D receptor (VDR) in skeletal muscle tissue has been questioned [15]. However, recent data strongly indicate that VDR is expressed in C2C12 myoblasts and myotubes [16, 17], in murine skeletal muscle [18], and *in situ* detection of VDR in human skeletal muscle points towards a role of vitamin-D on muscle function [19, 20]. In addition, VDR has been located in skeletal muscle cells that promote *de novo* protein synthesis [21]. Addition of the active form of vitamin-D (1,25-dihydroxyvitamin D_3_ (1,25(OH)_2_D_3_)) to C2C12 myotubes has been shown to increase skeletal muscle protein synthesis and Akt/mTOR-signaling [22], down-regulate the expression of Myostatin and increase the size of myosin heavy chain (MHC) type II positive myotubes [17]. Moreover, vitamin-D signaling has been reported to alter gene expression and increase C2C12 myotube size [23]. These findings support the hypothesis that vitamin-D has a direct positive effect on the contractile filaments and thus muscle strength. Furthermore, preliminary data from another exercise experiment in our lab suggest that resistance exercise increases mRNA expression of VDR and 25-hydroxy vitamin-D (25(OH)D) hydroxylase cytochrome p450 27B1 (CYP27B1), which is responsible for converting the inactive form of vitamin-D, 25(OH)D, to the active form, 1,25(OH)_2_D_3_, further supporting a positive role for vitamin-D in skeletal muscle.

Studies in humans have shown positive effects of concomitant intake of vitamin-D on muscle strength [24–29] and intramyonuclear VDR level [30] in vitamin-D insufficient individuals. Moreover, it has been shown that vitamin-D intake increases cross sectional area (CSA) of skeletal muscle fibers [30], the diameter and number of type II muscle fibers [31] and specifically type IIa fibers [32]. However, it appears that elderly vitamin-D insufficient people may benefit the most from vitamin-D intake [33] whereas young people might not [34].

Based on these findings we wanted to examine the hypothesis that vitamin-D is important for the hypertrophic response to resistance exercise; specifically whether serum vitamin-D levels may influence the muscular response to resistance training. UVB radiation from the sun is the primary source for vitamin-D production and is very low at northern latitudes from September to April [35]. The resulting seasonal fluctuations in serum vitamin-D levels [36] could influence muscle strength and function. If this is so, it is possible that low serum vitamin-D levels during the winter months could partly blunt the positive effect of resistance training, and in such case, vitamin-D supplementation could be important for maximizing improvements in skeletal muscle mass and strength during resistance training.

The aim of the present study was to investigate whether vitamin-D intake during 12 weeks of resistance training has an additive effect on muscle hypertrophy and strength. We hypothesized that intake of vitamin-D plus calcium would improve the outcome of three months of resistance training in healthy untrained individuals resulting in greater muscle strength and hypertrophy compared to a training control-group supplemented with calcium alone (placebo). Moreover, we hypothesized that resistance exercise would increase the mRNA expression of VDR and CYP27B1. The study included a group of young and a group of elderly individuals to elucidate a possible blunted hypertrophic response in the aging muscle.

## Materials and methods

### Participants

Healthy, sedentary young (aged 20–30 years) and elderly (aged 60–75 years) Caucasian men living within the local community in Copenhagen, Denmark, were recruited through newspaper and web advertisements. Exclusion criteria were: participation in resistance exercise during the preceding 6 months, intake of >5 μg vitamin-D one month prior to the trial period, body mass index (BMI) <18 or >30, smoking, history of serious knee injury, any medical condition or medication known to affect protein turnover, diagnosis or a family history of organic dysfunctions, cancer, illnesses that could affect the musculoskeletal system, or blood tests values outside normal range. During the trial period, the participants were told to refrain from increased exposures to UVB radiation through e.g. use of solarium or travel activity.

### Setting

The study took place at Bispebjerg Hospital, Copenhagen, Denmark (latitude of 56°N). Inclusion was continuous from November 17, 2010 to December 21, 2010 and the last subject completed the study on April 25, 2011. Thus, the study was conducted in a period of low UVB irradiation from sunlight. Participants were informed about the study protocol, the risks of tests and investigations, and their rights according to the Declaration of Helsinki II. The study protocol was approved by Research Ethics Committee Region Hovedstaden Committee F (H-2-2010-100) and by the Danish Data Protection Agency (2011-41-5965). All participants gave written informed consent. The study was registered at ClinicalTrials.gov (nr. NCT01252381).

### Randomization and blinding

Participants were continuously randomized by the envelope method. Young and elderly men were randomized separately. Allocation ratio was 1:1 for vitamin-D and placebo groups. The subjects were enrolled and randomized by the principal investigator. Personnel conducting the scans, analyzing CSA, measuring isometric muscle strength, taking biopsies and blood samples, analyzing muscle fiber type composition and performing RT-qPCR analyses, were all blinded with respect to allocation.

### Interventions

#### Vitamin-D and calcium supplementation

The participants were randomly assigned (double-blinded) to a vitamin-D group receiving 48 μg (1920 IU) vitamin-D_3_ (cholecalciferol)/day plus 800 mg calcium/day or a placebo group receiving 800 mg calcium/day alone for 16 weeks. All participants were instructed to take two tablets orally each day. The vitamin-D-group received one tablet containing 10 μg vitamin-D_3_ + 400 mg calcium (Silver, Unikalk, Frederiksvaerk, DK) and one tablet containing 38 μg vitamin-D_3_ + 400 mg calcium (Mega, Unikalk, Frederiksvaerk, DK), thus reaching the maximum advisable daily dose (50 μg, 2000 IU) by the Danish Health and Medicines Authority. The placebo-group received an equivalent dose of calcium by consuming two calcium tablets (400 mg per tablet) (Basic, Unikalk, Frederiksvaerk, DK). The two tablets were taken simultaneously at the same time each day together with a meal. Tablets containing vitamin-D plus calcium or calcium alone were indistinguishable and only labeled with the name of the manufacturer.

### Training protocol

Participants were subjected to 36 training sessions (12 weeks with 3 sessions/week) consisting of 5–10 min warm-up on cycle ergometers followed by resistance training exercises of the lower extremities performed in commercial knee extension and leg press devices (Technogym, Super Executive Line, Gambettola, Italy). All sessions were supervised. Progressive loading levels were monitored continuously and adjusted throughout the entire training period to maintain muscle loading at the intended values.

Prior to commencement of training, 1-repetition maximum (1RM) strength was estimated from a 5-repetition-maximum (5RM) test [37]. The 5RM test was performed every two weeks, to ensure that the training load was relative to the strength progression throughout the training period. During the first 6 training sessions, participants completed 3 sets of 12–15 repetitions at 65–70 % of 1RM. During session 7–12, participants performed 3 sets of 10–12 repetitions at 70–75 % of 1RM, increasing to 4 sets at 70–75 % of 1RM during session 13–18. From session 19 and onwards, participants performed 5 sets with training load progressing from 8–10 repetitions at 75–80 % of 1RM in session 19–27 to 6–8 repetitions at 80–85 % of 1RM in session 28–36 [38]. The exercises were performed in a moderate slow, controlled manner with 1–2 s in the concentric- and eccentric phase with a rest of 1–3 min between sets. Exercise compliance (sets, repetitions, and load) was calculated from daily exercise records completed by the instructors at each training session. Participants were informed that a mean attendance of less than 2 training sessions per week resulted in exclusion. All adverse events associated with the training intervention were recorded.

### Analyses

#### CSA of quadriceps muscle

CSA of quadriceps muscle was measured pre (week 0) and post (week 12) training intervention. Magnetic Resonance Imaging (MRI) (3.0 T, T1-weighted, Radiographic department, Bispebjerg Hospital, Copenhagen, Denmark) was conducted to obtain six images starting at the tibia plateau and proceeding proximally to the mid/proximal femur. Each slice was 6 mm thick and the distance between each slice was 54 mm. The slice tangential to the tibia plateau was used as an anatomical marker (first slice) and the subsequent slices were numbered slice-by-slice proximally. The fourth axial slice of the thigh was selected for further analysis. To avoid any effect of body fluid disturbances directly related to the preceding exercise session, the MRI-scans were performed no sooner than 48 h post exercise and before the biopsies were taken. CSA images of both legs were analyzed for changes from pre to post training by manually drawing margins of the quadriceps muscle (Osirix, open-source 32 bit, Pixmeo SARL, Geneva, Switzerland) and a mean of both legs was calculated. Images from one participant were analyzed in succession to ensure uniform measure technique. Analyses were performed blinded according to trial group and time point. Whether the pre- or post picture was analyzed first was randomized.

### Isometric muscle strength

All participants were familiarized with the strength test at inclusion in order to reduce any learning effects. Isometric knee extension peak torque of both legs was measured pre and post 12 weeks training with the participants sitting in a custom-made rigid chair with hips and knees flexed to 90°. To avoid movement or unwanted contribution of other muscles, participants were strapped at the hip and the abdomen. A leg cuff, connected to a strain gauge (Bofors KRG-4, Bofors, Sweden) through a rigid steel rod perpendicular to the lower leg, was mounted on the lower leg just above the medial malleolus. The signal from the strain gauge was amplified (Noraxon Telemyo 2400 T G2) and sent to a computer to analyze the greatest force (N) for each participant (Myoresearch, XP Master Edition 1.07.25). Lever arm was defined as the distance from the joint line of the knee to the middle of the leg cuff. After a 5-min warm-up on a cycle ergometer and 2–3 submaximal pre-test-measurements, each leg was measured 3 times with 2 min rest between each measurement. Participants were randomized to start with either the left or the right leg.

On the basis of the quadriceps CSA and isometric strength measurement, isometric strength/CSA was calculated as an expression of muscle quality.

### Serum 25(OH)D concentrations

Blood samples were obtained at baseline (week −4) and after 0, 2, 6 and 12 weeks of resistance training. Serum 25(OH)D concentrations were determined using an ELISA-kit (Elecsys, kit ref. 05894913190, Roche Diagnostics A/S, Denmark) measuring both 25(OH)D_2_ and 25(OH)D_3_ on a Cobas e411 (Roche Diagnostics A/S, Denmark) at the Department of Clinical Biochemistry, Bispebjerg Hospital.

### Muscle biopsy

A biopsy from the lateral part of the vastus lateralis muscle from either the left or the right leg was obtained before the training started (week 0) and after the last training session (week 12). At week 12, two biopsies were taken; 4 h (TR+4h) and 48 h (TR+48h) after the last exercise session, respectively. The biopsies were obtained using a 4-mm biopsy needle (Bergström, Stockholm, Sweden). Briefly, the skin was shaved and disinfected before local anesthetization (1 % lidocaine). An incision was made through which the muscle biopsy was taken. Afterwards, the incision was strapped with SteaStrips and covered with waterproof plaster. The muscle specimen was quickly freed from any visible blood, fat or connective tissue and subsequently divided in two; one piece was mounted in Tissue-Tek and frozen in precooled isopentane, the other piece was frozen in liquid nitrogen. Both pieces were stored at −80 °C until further analysis.

### Fiber type analysis

Cross sections (10 μm) were cut from the muscle specimen mounted in Tissue-Tek in a cryomicrotome (HM 560, Microm, Germany) at −20 °C and stained for myofibrillar ATPase at pH 9.4 after both alkaline (pH 10.3) and acid (pH 4.3 and 4.6) pre-incubations as previously described [39]. All samples were stained in the same batch to avoid inter-assay variations. Muscle fiber type and size were analyzed in a blinded fashion. Only true horizontal fibers were analyzed for size. Thereby, slightly fewer fibers could be analyzed for size (202 ± 7 fibers per biopsy, mean ± SD) than for type (204 ± 4 fibers per biopsy). As previously described [38], muscle fibers were characterized as type I, IIa, or IIx based on the ATPase staining pattern. Tema Image-analyses System (Scanbeam, Denmark) was used to analyze the cross sections for muscle fiber type and size. The fiber type distributions and mean fiber CSA were calculated. Muscle fiber morphology analysis included determination of fiber type % and mean CSA. Type IIa and IIx fiber results were pooled to yield the combined type II fiber area.

### Measurement of mRNA

The mRNA expression of VDR, CYP27B1 and Myostatin was measured using real-time reverse transcriptase polymerase chain reaction (RT-PCR). Approximately 20 mg of muscle tissue was homogenized in 1000 μl TriReagent (Molecular Research Center, RT 118) using a FastPrep 24 (MP Biomedicals), with muscle tissue placed in a 2 ml microvial with a screw cap (BioSpec) containing one silicium-carbide crystal and five 2.3 mm steel beads. Thereafter, 1-Bromo-3-chloropropane (BCP) was added to the homogenized sample to separate the sample into an aqueous and an organic phase. The RNA was precipitated from the aqueous phase using isopropanol, washed with ethanol, and dissolved in RNase-free water. The RNA was then re-precipitated using sodium acetate (NaAc), washed in ethanol, and dissolved in RNase-free water. RNA concentrations and purity were determined by spectroscopy, and RNA quality was ensured by gel electrophoresis.

Synthesis of complementary DNA (cDNA) was performed using Omniscript reverse transcriptase (Qiagen, Hilden, Germany). The cDNA synthesis was performed on 500 ng of muscle RNA. cDNA was diluted 20 times in TE-buffer containing 1 ng/μl Salmon DNA. For each target, 5 μl of diluted cDNA was amplified (25 μl total volume) in Quantitect SYBR Green Master Mix (Qiagen) with specific primers (0.1 μM each, Table 1) on a real-time PCR machine (Stratagene MX3005P, Agilent Technologies, USA). The thermal profile was set to 95 °C for 10 min, followed by 50 cycles of amplification; 95 °C for 15 s., 58 °C for 30 s., 63 °C for 90 s. Signal intensity was measured at the 63 °C step, and the threshold cycle (C_t_) values were related to a standard curve made from the cloned PCR product or corresponding synthetic oligonucleotides. Specificity of the PCR product was confirmed by a melting curve analysis after the last amplification cycle (55 °C to 95 °C).

**Table 1:** Primers used for real-time reverse transcription PCR

Target	Sense primer	Antisense primer	Ref. sequence
RPLP0	GGAAACTCTGCATTCTCGCTTCCT	CCAGGACTCGTTTGTACCCGTTG	NM_053275.3
GAPDH	CCTCCTGCACCACCAACTGCTT	GAGGGGCCATCCACAGTCTTCT	NM_002046.4
VDR	CAGGCCCAACTCCAGACACACT	ATCCAGATTGGAGAAGCTGGACGA	NM_000376.2
CYP27B1	AAGCGCAGCTGTATGGGGAGAC	GCTCAGGCTGCACCTCAAAATG	NM_000785.3
Myostatin	TTCGTCTGGAAACAGCTCCTA	GGAGTCTCGACGGGTCTCAA	NM_005259.2

**Fig. 1:**
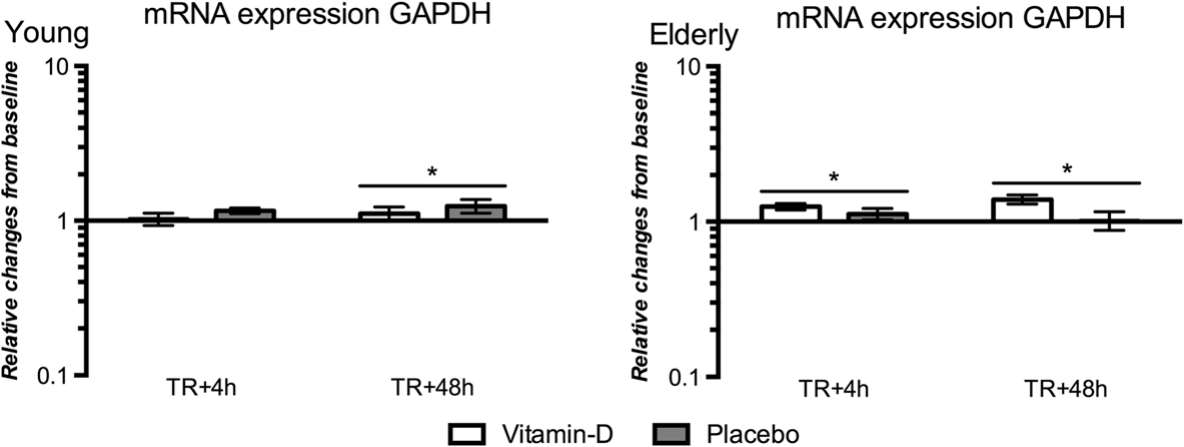
GAPDH mRNA expression normalized to RPLP0. Shown as fold changes post 12 weeks training compared to pre training on logarithmic scale at 4 h (TR+4h) and 48 h (TR+48h) after the last exercise session. Results are shown as geometric mean ± back-transformed SEM. * different from pre training (*p* < 0.05)

Ribosomal protein large P0 (RPLP0) was chosen as housekeeping gene. As a control, Glyceraldehyde 3-phosphate dehydrogenase (GAPDH), another often constitutively expressed mRNA, was normalized to RPLP0 (Fig. 1). Changes were seen in GAPDH mRNA expression when normalized to RPLP0. Most likely this was due to changes in GAPDH mRNA expression which possibly can occur due to 12 weeks resistance training. However, a change in mRNA expression such as that of the housekeeping genes in Fig. 1 would not affect normalization of the targets of interest (VDR, CYP27B1 and Myostatin) to a degree that would explain the differences we see in mRNA expression of these gene targets.

### Statistical analyzes

Based on a previously published article on the effect of heavy and light load resistance training, respectively, on muscle growth [40] where the heavy training was equivalent to the training used in this study a power calculation was carried out to estimate how many subjects should be included to be able to detect a significant difference between the desired outcome parameters. With a power of 0.8 and a significance level at 0.05 we calculated that a group size of 10 was needed to detect the expected increase in CSA and strength.

Analyzes were conducted per protocol and separately for the young and elderly men to explore the effect of vitamin-D intake vs. placebo. Furthermore, young vs. elderly was compared to test differences between age groups. Data were checked for normality and found to be normally distributed. Inclusion characteristic results were analyzed using an unpaired t-test. The results of serum 25(OH)D concentrations, muscle fiber type composition and gene expression were analyzed by a two-way analysis of variance (ANOVA) with repeated measures for time. Serum 25(OH)D concentrations and muscle fiber type composition are presented as mean ± standard error of the mean (SEM). Quadriceps muscle CSA, isometric muscle strength and isometric strength/CSA were calculated as relative changes from pre to post training and thus presented as mean percentage-changes ± SEM. Pearson correlation analyzes were conducted using Prism 6 (GraphPad Software Inc., CA, USA). Muscle fiber type data are shown as mean ± SEM. Results of mRNA data post training (TR) from biopsies taken 4 h (TR+4h) and 48 h (TR+48h) after the last exercise session are reported relative to the pre training (week 0) values. The mRNA data are shown as geometric (Geo) means ± back-transformed SEM. All mRNA data were log-transformed and normalized to RPLP0.

Unless otherwise stated, data were analyzed by a two-way ANOVA with repeated measures for time. When significant changes were found by ANOVA testing, a Student-Newman-Keuls post-hoc test was performed. Level of significance was *p* < 0.05. For a p-value between 0.05 and 0.1 a tendency was discussed. Statistical analyzes were conducted using SigmaPlot software version 11.0 unless otherwise stated.

## Results

**Fig. 2:**
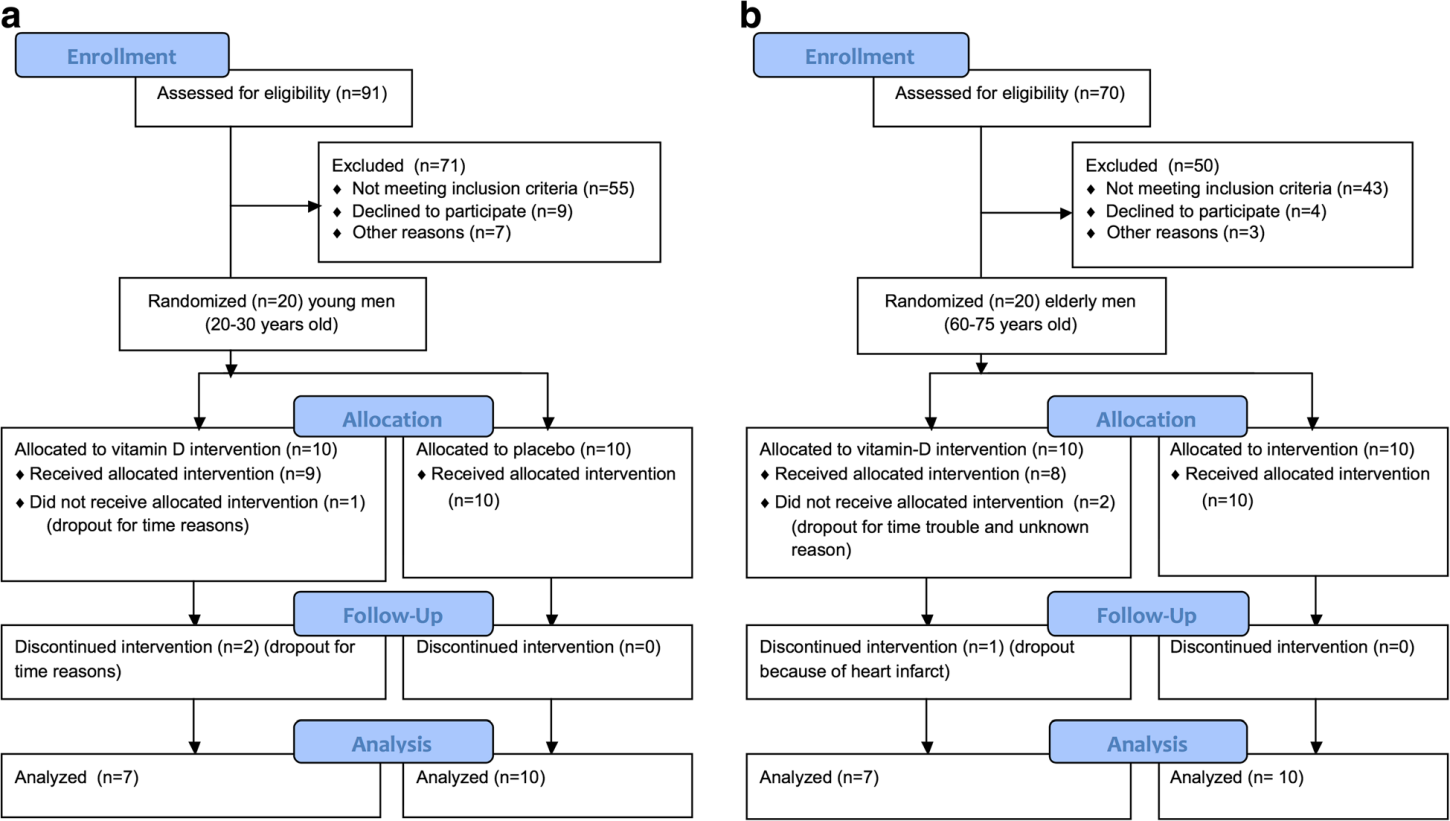
Flowchart showing **a** young and **b** elderly subjects from first contact to end of study

Healthy untrained men, 20 young and 20 elderly, were included in the study. 6 participants (3 young and 3 elderly), all from the vitamin-D-groups, dropped out. Reasons for dropping out were time consumption (*n* = 4), health problem not related to the project (*n* = 1) and unknown reason (*n* = 1). The remaining 34 participants were included in the final per protocol analysis (Fig. 2). MRI scans were not performed in one elderly from the placebo group because of a metal object in his calf.

### Baseline characteristics

With the exception of a greater quadriceps CSA and a lower isometric strength/CSA in the vitamin-D group compared to the placebo group of elderly men, no significant differences between the vitamin-D- or placebo groups were observed for the baseline characteristics (Table 2).

**Table 2:** Inclusion characteristics. Baseline characteristics (Mean ± SD) of young and elderly subjects in vitamin-D and placebo group, respectively

Young men	Vitamin-D (*n* = 7)	Placebo (*n* = 10)	Group difference (*p*-value)
Age, years	23.3 ± 2.0	22.4 ± 1.8	0.39
Height, cm	181.6 ± 6.3	181.1 ± 5.5	0.85
Weight, kg	77.9 ± 11.4	75.6 ± 9.1	0.66
Body mass index, kg/m^2^	23.6 ± 3.6	23.0 ± 2.3	0.69
CSA, cm^2^	57.9 ± 9.7	59.0 ± 4.8	0.78
Isometric muscle strength, Nm	215.6 ± 30.7	209.5 ± 30.5	0.71
Strength/CSA, Nm/cm^2^	3.82 ± 0.92	3.55 ± 0.47	0.45
Elderly men	Vitamin-D (*n* = 7)	Placebo (*n* = 10)	Group difference (*p*-value)
Age, years	67.1 ± 2.9	66.6 ± 4.2	0.78
Height, cm	178.5 ± 1.9	178.8 ± 6.7	0.93
Weight, kg	84.8 ± 5.7	80.4 ± 9.3	0.32
Body mass index, kg/m^2^	26.6 ± 1.8	25.1 ± 1.1	0.11
CSA, cm^2^	53.7 ± 2.7	47.0 ± 7.3	0.047
Isometric muscle strength, Nm	154.6 ± 22.0	168.3 ± 35.3	0.41
Strength/CSA, Nm/cm^2^	2.89 ± 0.45	3.46 ± 0.49	0.03

### Training compliance

The participants completed 12 weeks of training with a mean training frequency of 2.8 ± 0.2 times (mean ± SD) per week (with no subjects <2 times/week). For the young men, the mean training frequencies were 2.9 ± 0.2 and 2.8 ± 0.2 times per week in the vitamin-D and the placebo group, respectively. For the elderly men, the mean training frequency was 2.7 ± 0.2 times per week in both the vitamin-D and the placebo group.

### Adverse events

There were no serious injuries or adverse events associated with the exercise program, but symptoms occurred in 6 young subjects (3 from the vitamin-D group and 3 from the placebo group) and 9 elderly subjects (4 from the vitamin-D group and 5 from the placebo group). The symptoms were usually located to the knee joint but also pain located in the neck, hip, groin and lumbar back was recorded. These participants continued exercising with a lower intensity for a few days to 3–4 weeks. No dropouts were related to injuries or pain.

### Serum 25(OH)D concentrations

**Fig. 3:**
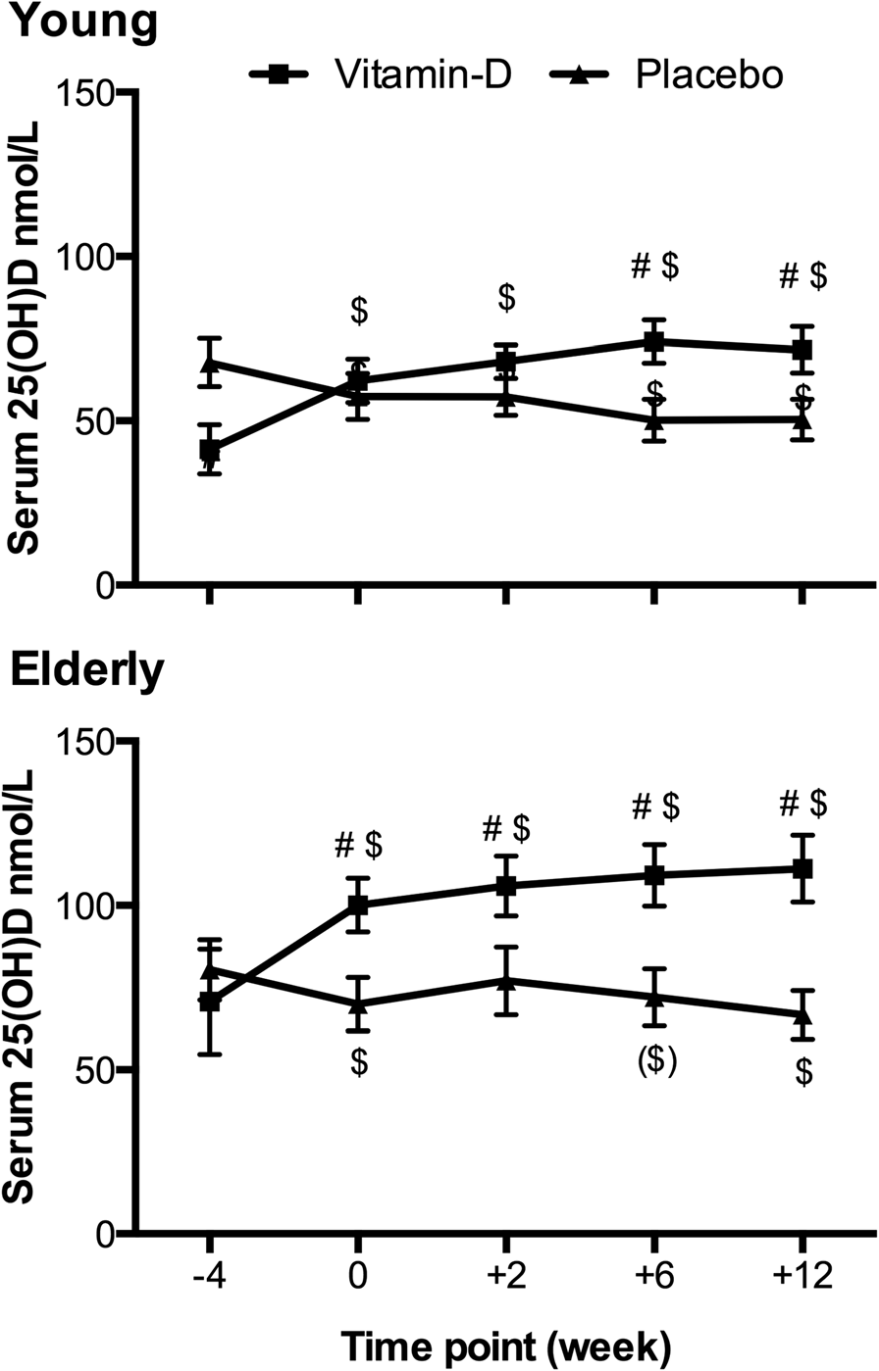
Serum 25(OH)D concentrations. Mean serum 25(OH)D concentrations ± SEM for young and elderly vitamin-D and placebo groups. Week 0 denote start of training period. # different from placebo (*p* < 0.05). $ different from week −4 (*p* < 0.05). () denote tendency (0.05 < *p* < 0.10)

Vitamin-D and calcium intake commenced 4 weeks before the training started (week -4). In the young vitamin-D group, the serum concentrations of 25(OH)D were significantly increased at time-point 0, 2, 6 and 12 weeks as compared to week -4 (Fig. 3). At time-point 6 and 12 weeks, the serum 25(OH)D concentrations in the young vitamin-D group were significantly higher than the placebo group. In the elderly vitamin-D group, the serum 25(OH)D concentrations were significantly increased at time-point 0, 2, 6 and 12 weeks as compared to week -4. At the same time-points (week 0, 2, 6 and 12 weeks), the 25(OH)D concentrations were significantly higher than that of the elderly placebo group.

### CSA of quadriceps muscle

**Fig. 4:**
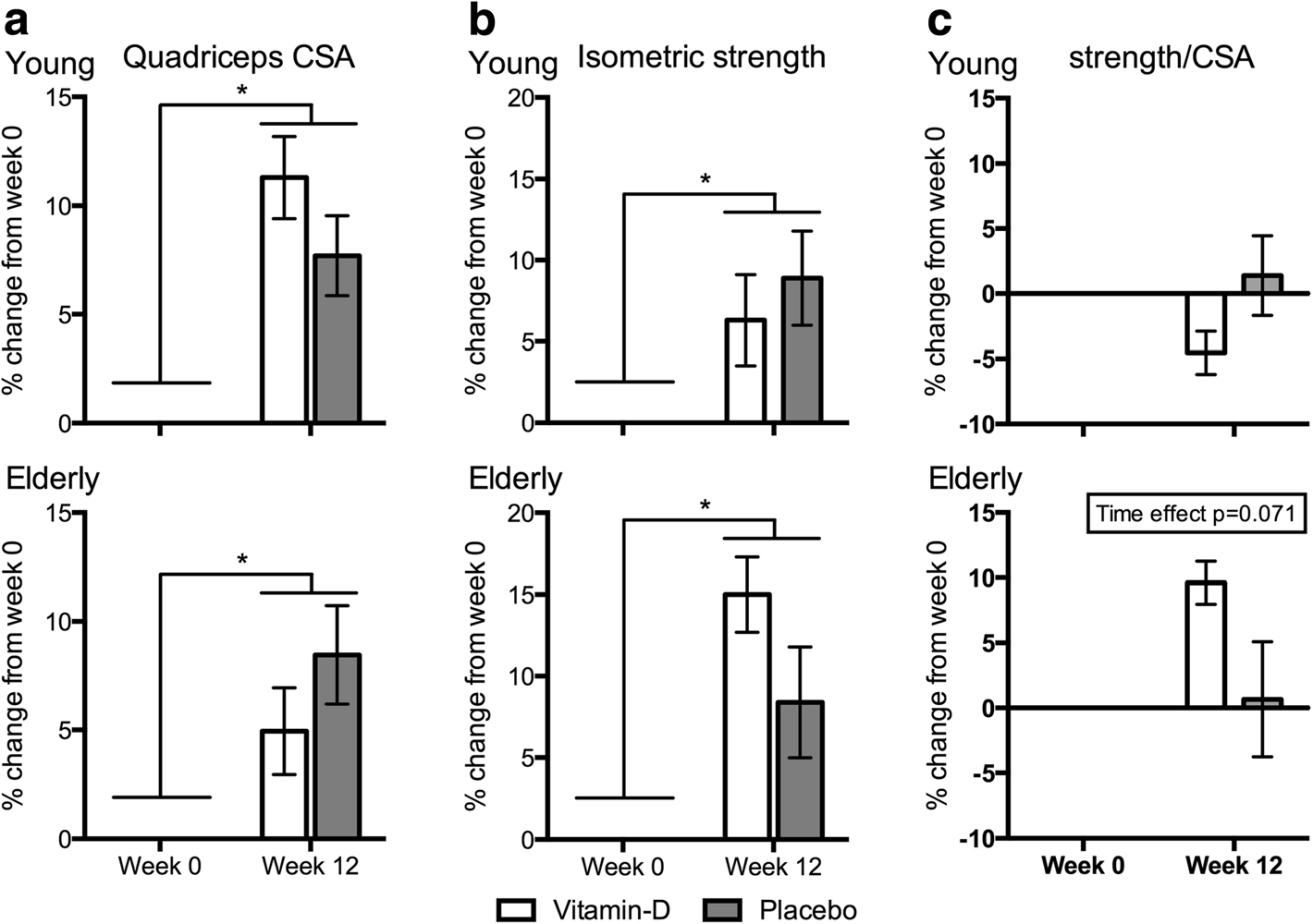
CSA, Isometric strength and strength/CSA of Quadriceps muscle. Change in **a** CSA, **b** isometric strength and **c** strength/CSA of quadriceps muscle for young and elderly vitamin-D and placebo groups, respectively. Data shown as mean percentage change from week 0 ± SEM. * different from week 0 (*p* < 0.05)

All four groups had significant gains in quadriceps muscle CSA after 12 weeks of resistance training (Fig. 4a). The changes were 11.3 ± 1.9 % and 7.7 ± 1.8 % for the young men and 4.9 ± 2.0 % and 8.5 ± 2.8 % for the elderly men in the vitamin-D and placebo group, respectively. There were no significant differences in CSA gains between the vitamin-D and placebo groups. When comparing across age groups no significant differences were seen in CSA gains (Additional file 1: Table S1).

### Isometric muscle strength

All four groups demonstrated significant increases in quadriceps muscle isometric strength after 12 weeks of resistance training (Fig. 4b). The changes were 6.3 ± 2.8 % and 8.9 ± 2.9 % for the young men and 15.0 ± 2.3 % and 8.4 ± 3.4 % for the elderly men in the vitamin-D and placebo group, respectively. There were no significant differences in isometric muscle strength gains between the vitamin-D and placebo groups. Comparing young vs. elderly men, no significant differences were seen in the isometric strength gains (Additional file 1: Table S1).

### Isometric strength/CSA

No significant increase in the isometric strength/CSA was seen after 12 weeks resistance training in young or elderly, and no significant differences was seen between the vitamin-D and placebo groups (Fig. 4c). However, a tendency towards a main effect of time was seen in the elderly. Comparing young and elderly, the elderly vitamin-D group had a greater increase in the % change of isometric strength/CSA compared to the young vitamin-D group (Additional file 1: Table S1).

### Correlation

No correlations were found between weighted mean serum 25 (OH) D concentrations (during the 12 week training period) and mean percentage changes of quadriceps muscle CSA, isometric strength or strength/CSA (Additional file 2: Figure S1).

### Muscle fiber type morphology

**Fig. 5:**
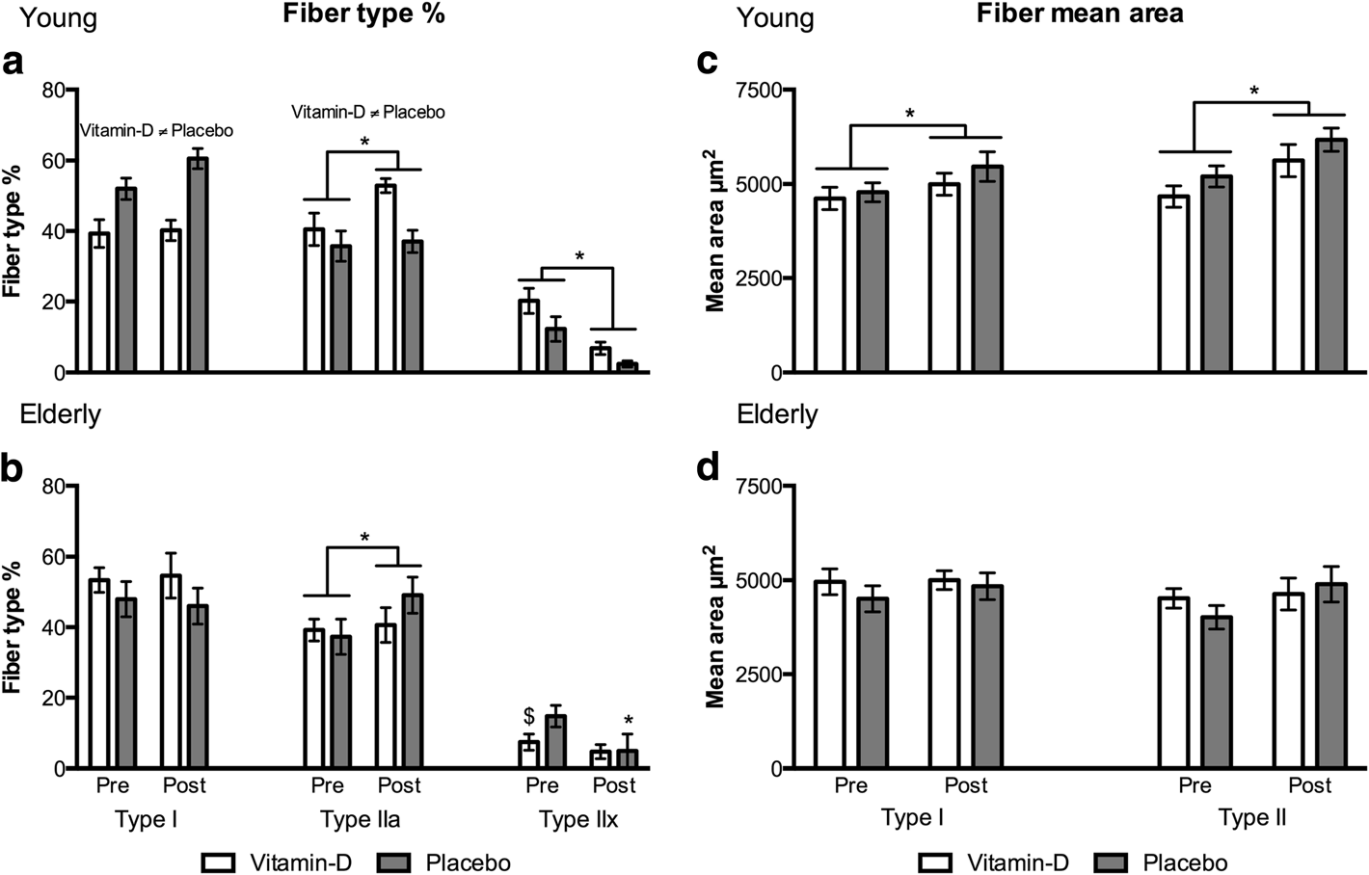
Fiber type percentage and mean area **a** and **b** Fiber type (I, IIa and IIx) percentage and **c** and **d** fiber type (I and II) mean area for young and elderly vitamin-D and placebo groups, respectively. Shown as mean fiber type percentage ± SEM at pre training (week 0) and post training (week 12). * different from pre training (*p* < 0.05). $ different from Placebo group (*p* < 0.05)

By 12 weeks resistance training, the percentage of type IIa fibers increased and the percentage of type IIx fibers decreased in both young and elderly (Fig. 5a and 5b). However, the decrease in type IIx for the elderly was only seen in the placebo group. Only in the young men was a significant increase in fiber type II and type I mean area seen from pre to post training (Fig. 5c and 5d). In the elderly, no increase in fiber size was seen. For the elderly, the p-value for an ANOVA time effect on fiber type II mean area was 0.074. Comparing young vs. elderly for the % change in muscle fiber type percentage and mean area from pre to post training no differences were seen between age groups, but in the young subjects the vitamin-D group had a greater increase in type IIa percentage compared to the placebo group (Additional file 1: Table S1).

### Expression of mRNA

All mRNA data were normalized to the pre training basal mRNA levels. Thus, the mRNA data expresses the accumulated effect of training (TR) plus the acute effect after the last training session measured 4 h (TR+4h) and 48 h (TR+48h) post exercise. Importantly, this provided the opportunity to explore the different mRNA expression patterns after 12 weeks of vitamin-D supplementation and resistance training.

**Fig. 6:**
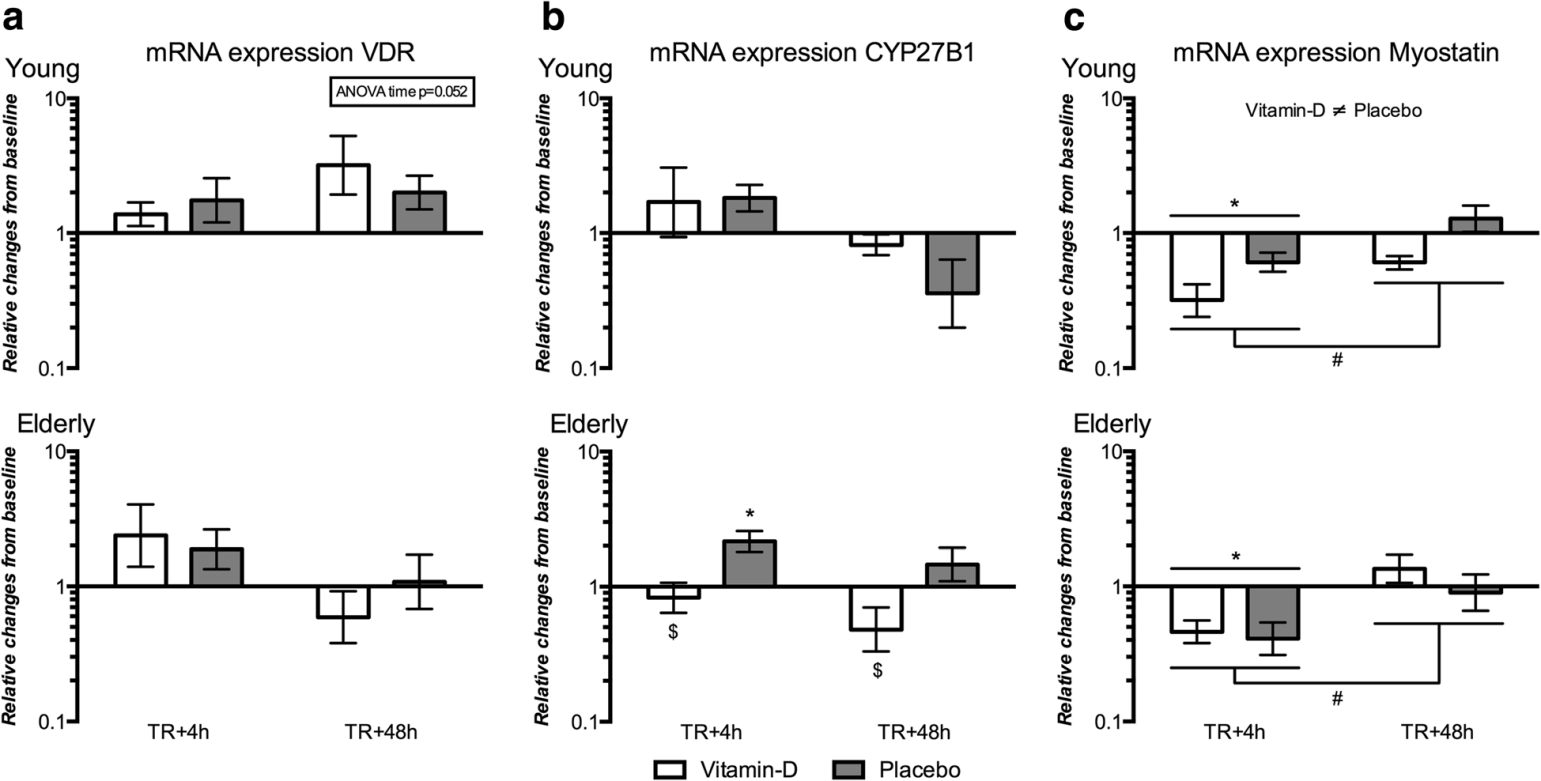
VDR, CYP27B1 and Myostatin mRNA expression. mRNA expression for **a** VDR, **b** CYP27B1 and **c** Myostatin shown as fold changes post 12 weeks training compared to pre training on logarithmic scale at 4 h (TR+4h) and 48 h (TR+48h) after the last exercise session. Results are shown as geometric mean ± back-transformed SEM. * different from pre training (*p* < 0.05). $ different from placebo (*p* < 0.05)

No difference in VDR mRNA expression was seen for any time point or between vitamin-D and placebo groups for either the young or the elderly men (Fig. 6a). For the elderly, a tendency was seen towards an increased mRNA expression of VDR at TR+4h compared to TR+48h (*p* = 0.069). Comparing young and elderly, no differences in VDR mRNA expression were seen (Additional file 3: Table S2).

In elderly, CYP27B1 mRNA expression at TR+48h was significantly lower in the vitamin-D compared to the placebo group (Fig. 6b). In the elderly placebo group at week 12, CYP27B1 mRNA expression was significantly increased when measured 4 h post resistance training (TR+4h) compared to pre training and compared to the vitamin-D group at the same time point. No significant change in CYP27B1 mRNA expression was seen in the young men, but it should be noted that a tendency towards an increase was seen for TR+4h compared to TR+48h (*p* = 0.074). In the placebo group, comparing young and elderly a greater CYP27B1 mRNA expression was seen in elderly at 48 h post training (TR+48h) (Additional file 3: Table S2).

Myostatin mRNA expression was decreased 4 h post training (TR+4h) compared to pre training and compared to 48 h post training (TR+48h) for the young and the elderly men, respectively (Fig. 6c). A difference in Myostatin mRNA expression was seen between the vitamin-D and placebo groups for the young men when measured post training. This difference was not time point specific. No differences in Myostatin mRNA expression were seen when comparing young vs. elderly in the vitamin-D and placebo group, respectively (Additional file 3: Table S2), but a main effect of time was seen with Myostatin mRNA expression at TR+4h being lower than pre training levels and TR+48h being greater than TR+4h.

## Discussion

The current study explores how vitamin-D intake influences the muscular improvement with 12 weeks resistance training in healthy untrained young and elderly men. Increased serum 25(OH)D concentrations were achieved in both the young and the elderly men with intake of 48 μg vitamin-D_3_/day, and the serum concentrations of 25(OH)D were higher in the vitamin-D groups compared to the control groups of young and elderly subjects, respectively. Increases in isometric muscle strength, muscle hypertrophy, and improved muscle fiber type morphology were seen after 12 weeks of progressive resistance training. But no differences in muscle strength or hypertrophy were found between the vitamin-D and placebo groups in either the young or the elderly men. However, the elderly vitamin-D group had a greater improvement in muscle quality, measured as strength/CSA, compared to the young vitamin-D group. Importantly, we found that 12 weeks of vitamin-D supplementation combined with resistance training in the young men lead to a greater change in Type IIa muscle fiber percentage and decreased the mRNA expression of Myostatin, which to our knowledge has not been reported before.

So far, three RCT studies have shown no additive effect of vitamin-D intake during training in elderly subjects [26, 41, 42]. However, the subjects in these studies were heterogeneous and training interventions suboptimal. Furthermore, the vitamin-D intake was only 10 μg (400 IU) to 40 μg (1600 IU)/day [26, 41, 42]. Thus, no overall conclusion on a possible additive effect of vitamin-D intake can be drawn on the basis of these studies. The lack of an additive effect of vitamin-D on skeletal muscle function in elderly with training could also reflect a blunted response in the aging muscle. Therefore, in designing this study it was important that vitamin-D intake and resistance training were optimal for increasing serum 25(OH)D, and strength and hypertrophy parameters, respectively, and that both young and old subjects were investigated.

Whereas studies have shown muscular improvements of vitamin-D plus calcium intake [24–29], three studies on vitamin-D without concomitant calcium supplementation showed no gain in muscle strength [43–45], suggesting that calcium is a prerequisite for the vitamin-D effect on skeletal muscle. Furthermore, control groups receiving calcium supplements alone have shown no increase in muscle strength. Thus, in the present study calcium was given to both groups. Calcium absorption is vitamin-D dependent. Therefore, it cannot be excluded that calcium plays a role in the skeletal muscles outcome, along with the proposed effect of vitamin-D.

The optimal dose of vitamin-D supplementation and optimal serum 25(OH)D concentrations are widely discussed, and recommendations regarding vitamin-D intake vary depending on age and grade of sufficiency, ranging from 10 μg/day in infants to 250 μg/day in adults with severe insufficiency [46]. But clearly, a dose–response relationship exists between daily dose and increments in serum 25(OH)D concentrations [41]. Serum 25(OH)D concentrations below 50 nmol/L are defined as vitamin-D insufficiency and concentrations around 90–100 nmol/L are reported to be optimal for lower extremity strength [47]. This study examined what effect supplemental vitamin-D intake might have on a population with sufficient 25(OH)D concentrations and not merely vitamin-D-insufficient subjects. Only the mean serum 25(OH)D for the young vitamin-D group at baseline (week -4) showed insufficiency (41.3 ± 7.5 nmol/L), whereas the elderly vitamin-D group had a baseline 25(OH)D concentration of 70.7 ± 16.0 nmol/L. The maximal increase in serum 25(OH)D concentration in absolute numbers, taking the variation into account, for the young and elderly were almost the same (32.8 nmol/L and 40.5 nmol/L, young and elderly, respectively). As the increase in serum 25(OH)D leveled off in the vitamin-D groups (Fig. 3) the baseline levels determined the maximal concentration achieved. Thus, it could be argued that the vitamin-D dose should have been further increased throughout the study. However, with the vitamin-D supplementation we did achieve serum 25(OH)D concentrations for the vitamin-D groups (maximal concentrations: 74.1 ± 6.6 nmol/L and 111.2 ± 10.2 nmol/L, young and elderly, respectively) that are expected to be close to optimal for improved muscle function [48], and a further increase in vitamin-D dose would have been impossible due to the recommendations from the Danish Health and Medicines Authority. Importantly, the mean serum 25(OH)D concentrations for the young and elderly placebo groups were significantly lower (50.4 ± 6.2 and 66.7 ± 7.4 nmol/L, respectively) and thus suboptimal. Therefore, with the intake of 48 μg/day in the present study, significant differences in serum 25(OH)D concentrations between the two groups, vitamin-D and placebo, were reached thus making it possible to examine potential differences in the response to resistance training due to concentrations of 25(OH)D. It should be noted that applying chromatography-mass spectrometry could be a more precise method of measuring serum 25(OH)D than the ELISA-kit used in the current study. However, all samples were measured at the same time with the ELISA-kit, which strengthens the data, and most importantly as vitamin-D_3_ is given the ELISA-kit includes measurement of 25(OH)D_3_.

Significant increases in CSA of quadriceps muscle in both young and elderly men were achieved. The increase in CSA in the young and in the elderly men is in line with the expected outcome of 12 weeks resistance training [40, 49–52]. A parameter that may affect the measurement of the CSA is the location of the analyzed slice from the MRI-scan. Therefore, in the present study the CSA of the quadriceps muscle was measured in the middle third of the thigh, as the greatest increase is known to occur in this location [50, 53]. The isometric strength of the quadriceps muscle increased in the young and the elderly after 12 weeks of resistance training. The increase for the young men (6.3 ± 2.8 % and 8.9 ± 2.9 %, vitamin-D and placebo, respectively) is slightly less than what has been reported previously in resistance training studies in healthy untrained young men [38, 40], whereas the increase in the elderly is in line with previous studies [50, 54]. Taking increase in CSA and isometric muscle strength into account, we believe that the training protocol applied has been optimal for gaining skeletal muscle mass and strength. In contrary to our hypothesis, no evidence for an additive effect of vitamin-D supplementation was seen on the CSA or isometric muscle strength of the quadriceps muscle in either the young or elderly men. It could be argued that the lack of effects of vitamin-D supplementation is due to lack of power in the analysis. However, when correlating the mean serum 25(OH)D concentration throughout the training period to quadriceps CSA or isometric strength no correlation was seen, indicating that serum 25(OH)D concentrations within sufficient concentrations do not affect skeletal muscle hypertrophy or strength improvements during resistance training. If muscle strength increases more than muscle CSA the muscle quality increases, indicating that the present muscle mass has improved the ability to exert muscle force. Thus, calculating muscle strength/CSA gives an estimate of the muscle quality. Comparing young and elderly, the elderly vitamin-D supplemented subjects improved their muscle quality more than the young vitamin-D group during the 12 weeks resistance training. This indicates that when performing resistance exercise, elderly subjects may benefit more from vitamin-D supplementation than young counterparts.

Muscle fiber type specific hypertrophy in response to resistance training of both slow-twitch type I fibers and fast-twitch type II fibers has been shown [55, 56], but most studies find hypertrophy of primarily type II fibers [38, 57]. After 12 weeks resistance training, a muscle fiber type switch occurred from IIx muscle fibers towards IIa muscle fibers (Fig. 5a and 5b). This is recognized as a normal response to resistance training. Again, this may indicate that the training intervention was effectively recruiting even the fastest motor units (type IIx fibers). In the elderly vitamin-D group, no decrease in fiber type IIx was seen. This is most likely caused by a small number of fiber type IIx at week 0, pre training, thus a significant decrease was difficult to detect. Increases in the mean area of type II fibers, but also type I fibers, in the young men also indicate an effective resistance training (Fig. 5c). However, the elderly men did not show any increase in type I or type II fiber mean area. From the ANOVA test of type II mean area in the elderly there was a tendency towards a time-point effect (*p* = 0.074), so it can be speculated that the training period in the present study was too short for the elderly. Thus, a longer training period for the elderly could lead to a significant increase in the mean area of type II fibers as seen in the young.

In response to vitamin-D, previous human studies have reported an effect on the mean area of type II fibers [31] and fiber type switch from IIx towards IIa [32]. In the young men, a significantly greater change in the percentage of type IIa fibers was seen in the vitamin-D group compared to the placebo group, indicating a positive effect from vitamin-D intake on the muscle fiber type morphology. However, as previously mentioned this difference was not translated into significantly greater muscle strength of the vitamin-D group compared to the placebo group. It should be noted that in the present study the improved muscle function has been measured as isometric muscle strength (force). Therefore, muscle power (force x speed) rather than muscle strength (force) could be a more functional measure for improvements in type II fibers if vitamin-D should have a selective effect on type II fibers as previously indicated [31, 32].

Myostatin mRNA expression was decreased 4 h after the last exercise session, which was an expected finding. Interestingly however, 12 weeks of vitamin-D supplementation decreased the Myostatin mRNA expression compared to the placebo group in the young men. Myostatin is a negative regulator of muscle mass, hence a decrease in Myostatin expression could induce a more positive balance between skeletal muscle protein synthesis and breakdown. Our finding on Myostatin mRNA level points towards a positive effect of vitamin-D intake on skeletal muscle mass and is in line with what has been seen in C2C12 myoblasts [17, 23]. Decreased Myostatin mRNA expression due to vitamin-D supplementation was only seen in the young men, thus an aging effect could exist but needs further investigation.

In contrary to our hypothesis, we did not see any increase in VDR mRNA expression in response to resistance training or in response to vitamin-D intake. Only the elderly showed a tendency towards an increased VDR mRNA expression 4 h after the last exercise session. The CYP27B1 mRNA expression was decreased in the elderly vitamin-D group post training. This could be a negative feedback mechanism from high serum concentrations of 25(OH)D (111.2 ± 10.2 nmol/L) for the elderly vitamin-D group. Thus, the active form of vitamin-D (1,25(OH)_2_D_3_) would be expected to be present in sufficient quantities and thereby decrease the need for the converting enzyme CYP27B1. The young vitamin-D group did not show a decrease in CYP27B1 mRNA expression post training. This may be explained by lower serum 25(OH)D concentrations (71.6 ± 7.1 nmol/L) compared to the elderly vitamin-D group.

## Perspectives

Without any exercise intervention, previous studies have shown a positive effect of vitamin-D intake on improved skeletal muscle function [24, 25, 27, 28]. However, these studies focused on vitamin-D-insufficient subjects, whereas in the present study the subjects were vitamin-D sufficient. Our primary findings, muscle strength and muscle hypertrophy, are in line with previous studies on elderly where the additive effect of vitamin-D and exercise training is examined [26, 41, 42], although the interventions in those studies have not been optimal for improving skeletal muscle function. In the present study, a progressive resistance training protocol was applied to attain an optimal gain in muscle strength and mass. Thus, any possible additive effect of vitamin-D on strength and hypertrophy could be overruled by the substantial effect of resistance training.

Previous studies seem to indicate that it is important to monitor vitamin-D levels in frail vitamin-D-insufficient subjects to maintain skeletal muscle function. This study indicates that high (vitamin-D groups) or low (placebo groups) vitamin-D levels within the sufficient range (above 50 nmol/L) results in the same muscle strength and mass gain from resistance training. Despite the lack of effect on our primary outcome, we still found that vitamin-D supplementation had a positive effect on muscle quality of the elderly and muscle fiber type morphology and Myostatin mRNA expression of the young subjects. This indicates that vitamin-D is involved in skeletal muscle remodeling and could be more important for certain age groups or vitamin-D insufficient subjects. Thus, studies have yet to elucidate to what extent vitamin-D intake could add to an improved skeletal muscle function in vitamin-D-insufficient and frail elderly when applied to resistance training or rehabilitation. Thus, vitamin-D intake in such subjects could be an important co-factor in fighting sarcopenia with resistance training.

## Conclusion

Optimal serum 25(OH)D concentrations for muscle function improvements were reached in vitamin-D supplemented healthy untrained young and elderly men. Despite this, no additive effect of vitamin-D supplementation was seen on the increase in quadriceps CSA or isometric strength from 12 weeks progressive resistance training in either the young or elderly men. However, vitamin-D intake during resistance training increased muscle quality in elderly men, and improved fiber type morphology and decreased Myostatin mRNA expression in young men. The latter may indicate an effect of vitamin-D on regulation of skeletal muscle mass, yet further investigations are required to explore this connection.

## Additional files

Additional file 1: Table S1. Hypertrophy and strength comparison Young vs. Elderly of % change from pre-values. p-values from two-way ANOVA test show outcome for main effects of Age and Vitamin-D, respectively, and interaction (Age x Vitamin-D). * different from Young. # different from Placebo. Data are shown as mean % change from pre (week 0) to post (week 12) training ± SEM. (DOCX 82 kb) Additional file 2: Figure S1. Correlation between Quadriceps ΔCSA, ΔIsometric strength, Δstrength/CSA and Serum 25(OH)D ΔCSA, ΔIsometric strength and Δstrength/CSA was calculated as the change from week 0 to week 12. Serum 25(OH)D was calculated as a weighed mean from week 0 to week 12. The correlation between serum 25(OH)D concentrations and changes of m. quadriceps CSA was for the young and elderly participants *r* ^2^ = 0.00 and *r* ^2^ = 0.01, respectively. The correlation between serum 25(OH)D concentrations and changes of isometric muscle strength was for the young and elderly participants *r* ^2^ = 0.01 and *r* ^2^ = 0.01, respectively. Whereas, the correlation between serum 25(OH)D concentrations and changes in strength/CSA was for the young and elderly participants *r* ^2^ = 0.01 and *r* ^2^ = 0.00, respectively. (DOCX 577 kb) Additional file 3: Table S2. mRNA comparison Young vs. Elderly within vitamin-D and Placebo group, respectively. p-values (two-way ANOVA with repeated measure for time) show the outcome for main effects of Age and Time, respectively, and interaction (Age x Time). § different from pre (week 0). $ different from TR + 4 h. * different from Young. Data are shown as logMean ± logSEM. (DOCX 66 kb)
