# Supplementary material for: Does vitamin-D intake during resistance training improve the skeletal muscle hypertrophic and strength response in young and elderly men? – a randomized controlled trial
Source: Nutr Metab (Lond). 2015 Sep 30;12:32. doi: 10.1186/s12986-015-0029-y (PMC4589960; doi:10.1186/s12986-015-0029-y)
Supplement: Additional file 1: Table S1. — Hypertrophy and strength comparison Young vs. Elderly of % change from pre-values. p-values from two-way ANOVA test show outcome for main effects of Age and Vitamin-D, respectively, and interaction (Age x Vitamin-D). * different from Young. # different from Placebo. Data are shown as mean % change from pre (week 0) to post (week 12) training ± SEM. (DOCX 82 kb) [file 12986_2015_29_MOESM1_ESM.docx]

|  | **Young** | | **Elderly** | | **p-values** | | |
| --- | --- | --- | --- | --- | --- | --- | --- |
| **Hypertrophy and strength**  *% change from pre training* | *Vitamin-D* | *Placebo* | *Vitamin-D* | *Placebo* | *Age* | *Vitamin-D* | *Age x Vitamin-D* |
| ΔCSA | 11.32 ± 1.90 | 7.68 ± 1.84 | 4.94 ± 2.00 | 8.46 ± 2.27 | 0.186 | 0.976 | 0.094 |
| ΔIsometric muscle strength | 6.31 ± 2.83 | 8.91 ± 2.88 | 14.96 ± 2.30 | 8.43 ± 3.38 | 0.193 | 0.527 | 0.148 |
| ΔStrength/CSA | -4.54 ± 1.65 | 1.38 ± 3.06 | 9.61 ± 1.64* | 0.66 ± 4.43 | 0.049 | 0.645 | 0.030 |
|  |  |  |  |  |  |  |  |
| **Fiber type**  *% change from pre training* |  |  |  |  |  |  |  |
| Type I percentage | 6.79 ± 10.61 | 22.07 ± 11.17 | 6.14 ± 14.54 | -2.45 ± 7.56 | 0.255 | 0.760 | 0.280 |
| Type IIa percentage | 39.08 ± 13.96^#^ | -1.59 ± 9.94 | 9.98 ± 18.75 | 31.08 ± 7.06 | 0.885 | 0.431 | 0.018 |
| Type IIx percentage | -65.34 ± 7.58 | -86.95 ± 5.31 | -55.70 ± 19.13 | -61.83 ± 12.15 | 0.146 | 0.242 | 0.510 |
| Type I mean area | 8.95 ± 5.05 | 7.35 ± 7.04 | 1.45 ± 14.51 | 9.45 ± 5.86 | 0.748 | 0.704 | 0.570 |
| Type IIa mean area | 16.12 ± 6.34 | 9.71 ± 8.53 | -6.45 ± 6.90 | 19.17 ± 8.28 | 0.415 | 0.237 | 0.054 |
| Type IIx mean area | 25.67 ± 8.56 | 26.49 ± 6.38 | 18.10 ± 15.11 | 23.68 ± 8.71 | 0.612 | 0.753 | 0.816 |

**Supplemental table 1 – Hypertrophy and strength comparison Young vs. Elderly** of % change from pre-values. p-values from two-way ANOVA test show outcome for main effects of Age and Vitamin-D, respectively, and interaction (Age x Vitamin-D). * different from Young. # different from Placebo. Data are shown as mean % change from pre (week 0) to post (week 12) training ± SEM.
