# Supplementary material for: Does vitamin-D intake during resistance training improve the skeletal muscle hypertrophic and strength response in young and elderly men? – a randomized controlled trial
Source: Nutr Metab (Lond). 2015 Sep 30;12:32. doi: 10.1186/s12986-015-0029-y (PMC4589960; doi:10.1186/s12986-015-0029-y)
Supplement: Additional file 2: Figure S1. — Correlation between Quadriceps ΔCSA, ΔIsometric strength, Δstrength/CSA and Serum 25(OH)D ΔCSA, ΔIsometric strength and Δstrength/CSA was calculated as the change from week 0 to week 12. Serum 25(OH)D was calculated as a weighed mean from week 0 to week 12. The correlation between serum 25(OH)D concentrations and changes of m. quadriceps CSA was for the young and elderly participants r 2 = 0.00 and r 2 = 0.01, respectively. The correlation between serum 25(OH)D concentrations and changes of isometric muscle strength was for the young and elderly participants r 2 = 0.01 and r 2 = 0.01, respectively. Whereas, the correlation between serum 25(OH)D concentrations and changes in strength/CSA was for the young and elderly participants r 2 = 0.01 and r 2 = 0.00, respectively. (DOCX 577 kb) [file 12986_2015_29_MOESM2_ESM.docx]

**
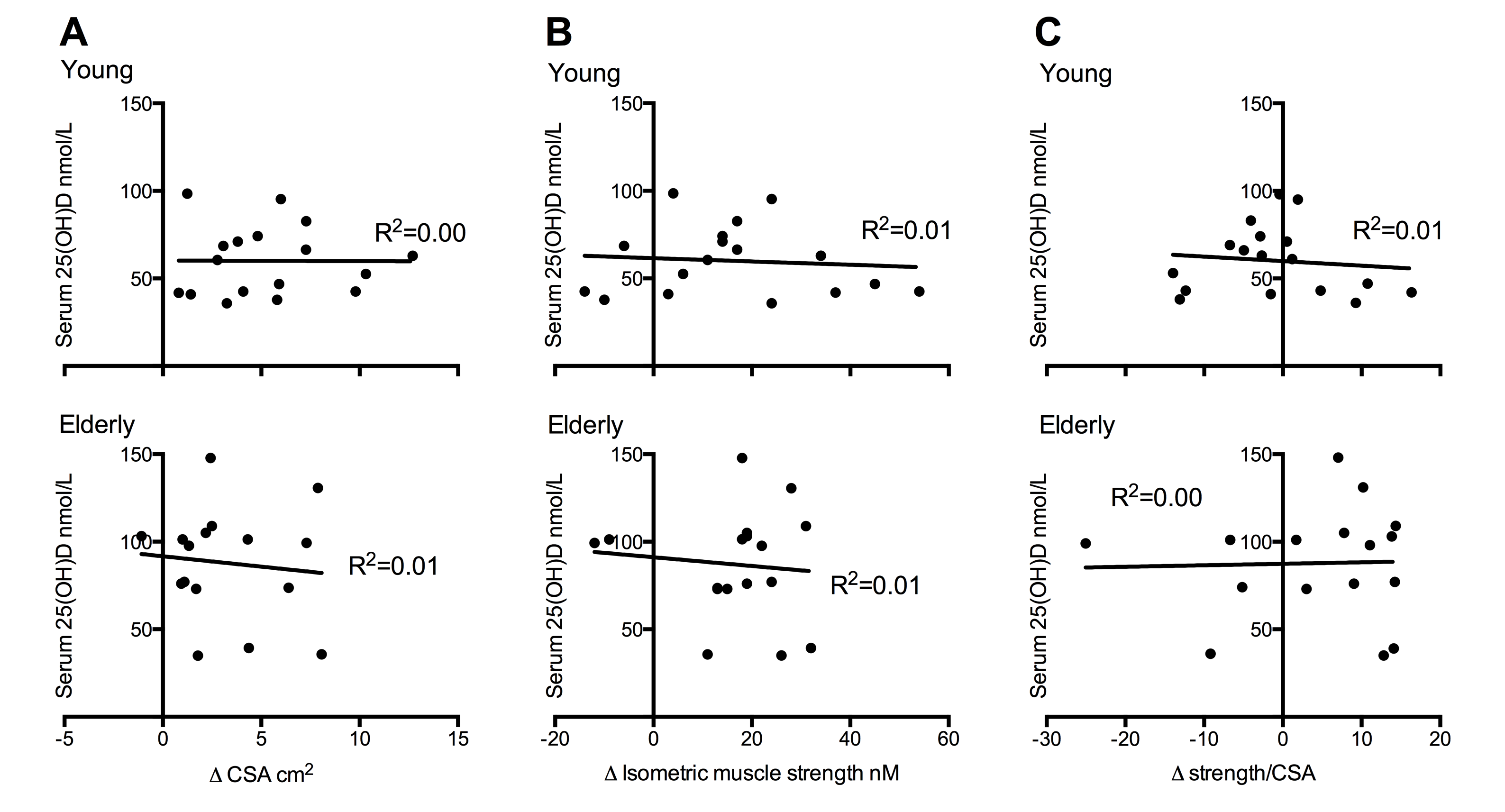
**

**Supplemental figure 1 - Correlation between Quadriceps ΔCSA, ΔIsometric strength, Δstrength/CSA and Serum 25(OH)D**

ΔCSA, ΔIsometric strength and Δstrength/CSA was calculated as the change from week 0 to week 12. Serum 25(OH)D calculated as a weighed mean from week 0 to week 12. The correlation between serum 25(OH)D concentrations and changes of m. quadriceps CSA was for the young and elderly participants r^2^=0.00 and r^2^=0.01, respectively. The correlation between serum 25(OH)D concentrations and changes of isometric muscle strength was for the young and elderly participants r^2^=0.01 and r^2^=0.01, respectively. Whereas, the correlation between serum 25(OH)D concentrations and changes in strength/CSA was for the young and elderly participants r^2^= 0.01 and r^2^=0.00, respectively.
