# Supplementary material for: Does vitamin-D intake during resistance training improve the skeletal muscle hypertrophic and strength response in young and elderly men? – a randomized controlled trial
Source: Nutr Metab (Lond). 2015 Sep 30;12:32. doi: 10.1186/s12986-015-0029-y (PMC4589960; doi:10.1186/s12986-015-0029-y)
Supplement: Additional file 3: Table S2. — mRNA comparison Young vs. Elderly within vitamin-D and Placebo group, respectively. p-values (two-way ANOVA with repeated measure for time) show the outcome for main effects of Age and Time, respectively, and interaction (Age x Time). § different from pre (week 0). $ different from TR + 4 h. * different from Young. Data are shown as logMean ± logSEM. (DOCX 66 kb) [file 12986_2015_29_MOESM3_ESM.docx]

|  | **Young** | | **Elderly** | | **p-values** | | |
| --- | --- | --- | --- | --- | --- | --- | --- |
| **Vitamin D** | *TR+4h* | *TR+48h* | *TR+4h* | *TR+48h* | *Age* | *Time* | *Age x Time* |
| VDR | 0.141 ± 0.097 | 0.503 ± 0.218 | 0.377 ± 0.193 | -0.232 ± 0.193 | 0.152 | 0.195 | 0.062 |
| CYP27B1 | 0.230 ± 0.256 | -0.086 ± 0.077 | -0.081 ± 0.111 | -0.322 ± 0.162 | 0.119 | 0.237 | 0.278 |
| Myostatin | -0.494 ± 0.120^§^ | -0.213 ± 0.049^$^ | -0.335 ± 0.088^§^ | 0.129 ± 0.105^$^ | 0.070 | <0.001 | 0.117 |
|  |  |  |  |  |  |  |  |
| **Placebo** |  |  |  |  |  |  |  |
| VDR | 0.244 ± 0.165 | 0.301 ± 0.126 | 0.273 ± 0.147 | 0.035 ± 0.201 | 0.540 | 0.061 | 0.445 |
| CYP27B1 | 0.259 ± 0.098 | -0.439 ± 0.247^§$^ | 0.334 ± 0.078^§^ | 0.165 ± 0.123* | 0.031 | <0.001 | 0.020 |
| Myostatin | -0.217 ± 0.072^§^ | 0.108 ± 0.097^$^ | -0.382 ± 0.117^§^ | -0.043 ± 0.135^$^ | 0.139 | <0.001 | 0.354 |

**Supplemental table 2 – mRNA comparison Young vs. Elderly** within vitamin-D and Placebo group, respectively. p-values (two-way ANOVA with repeated measure for time) show the outcome for main effects of Age and Time, respectively, and interaction (Age x Time). § different from pre (week 0). $ different from TR+4h. * different from Young. Data are shown as logMean ± logSEM.
